# A non-invasive, reference region-based method for quantification of cerebral blood flow and oxygen metabolism using oxygen-15 labeled gases and positron emission tomography

**DOI:** 10.22038/aojnmb.2025.81433.1578

**Published:** 2025

**Authors:** Hiroshi Ito, Masanobu Ibaraki, Ryo Yamakuni, Naoyuki Ukon, Shiro Ishii, Kenji Fukushima, Hitoshi Kubo, Kazuhiro Takahashi

**Affiliations:** 1 Department of Radiology and Nuclear Medicine, Fukushima Medical University, Fukushima, Japan; 2 Advanced Clinical Research Center, Fukushima Medical University, Fukushima, Japan; 3 Department of Radiology and Nuclear Medicine, Akita Research Institute of Brain and Blood Vessels, Akita, Japan; 4 Department of Radiological Sciences, School of Health Sciences, Fukushima Medical University, Fukushima, Japan

**Keywords:** Brain, CBF, CMRO_2_, OEF, PET

## Abstract

**Objective(s):**

Measurement of cerebral blood flow (CBF), cerebral blood volume (CBV), cerebral oxygen extraction fraction (OEF), and cerebral metabolic rate of oxygen (CMRO_2_) by positron emission tomography (PET) with oxygen-15 labeled gases is widely used for investigation into the pathophysiology of occlusive cerebrovascular disease. However, all methods for quantification of CBF, CBV, OEF, and CMRO_2_ by PET with oxygen-15 labeled gases require invasive arterial blood sampling. The present study developed a reference region-based method for quantification of CBF, CBV, OEF, and CMRO_2_ using PET and oxygen-15 labeled gases based on the steady-state method without invasive arterial blood sampling.

**Methods:**

The CBF, CBV, OEF, and CMRO_2_ were measured in patients with occlusive cerebrovascular disease by PET using ^15^O-labeled gases, C^15^O_2_, C^15^O, and ^15^O_2_, with the steady-state method. In the present method, the ratios of values in a brain region to the reference region for CBF, CBV, OEF, and CMRO_2_ were calculated without invasive arterial blood sampling.

**Results:**

Good correlations were observed for the ratios of values of the cerebral lesion to the reference brain region for CBF, CBV, OEF, and CMRO_2_ calculated by the present method as compared with those obtained by the steady-state method with arterial blood sampling, indicating its validity. Simulation studies showed that errors in estimated values calculated by the present method were negligibly small for both conditions of misery perfusion and matched hypoperfusion.

**Conclusion:**

A simple method for noninvasive quantification of CBF, CBV, OEF, and CMRO_2_ using PET and oxygen-15 labeled gases could be developed based on the steady-state method. This method can be used to investigate the pathophysiology of occlusive cerebrovascular disease.

## Introduction

 Measurement of cerebral blood flow (CBF), cerebral blood volume (CBV), cerebral oxygen extraction fraction (OEF), and cerebral metabolic rate of oxygen (CMRO_2_) by positron emission tomography (PET) with ^15^O-labeled carbon dioxide (C^15^O^2^), ^15^O-labeled carbon monoxide (C^15^O), or ^15^O-labeled oxygen (^15^O_2_) is widely used for investigation into the pathophysiology of several brain diseases, especially occlusive cerebrovascular disease (1-8). Decreased cerebral perfusion pressure due 

to major cerebral arterial occlusive disease causes cerebral autoregulatory vasodilatation to maintain CBF (stage I hemodynamic change). 

 Decreased cerebral perfusion pressure below the lower limit of cerebral autoregulation causes a decrease in CBF with an increase in OEF for maintenance of CMRO_2_ (stage II hemodynamic change). Although clinical assessment of stage II hemodynamic changes is widely performed using the more popular modality of single photon emission tomography (SPECT) to measure resting and acetazolamide-loaded CBF (9, 10), ^15^O-gas PET, which can directly detect elevated OEF, a specific hemodynamic index in stage II, is the most accurate and helpful modality (11).

 A significant problem with quantitative ^15^O-gas PET performed with various acquisition protocols is the need for invasive arterial blood sampling to measure arterial input function (AIF). While the risk of this blood sampling procedure in PET is not high (12), it is a barrier to widespread clinical use, especially with integrated PET/MRI systems, where the blood sampling procedure is complicated during the examination. Several ^15^O-gas PET studies have proposed methods to non-invasively estimate AIF: image-derived input function (IDIF), simultaneous estimation of the input function (SIME), and a combination of these methods (13-16). However, these non-invasive AIF estimation methods involve advanced, complicated image processing and mathematical modeling of dynamic PET images, making them impractical for clinical use and challenging to apply to the most widely used ^15^O-gas PET examination protocol, the steady-state method (17,18).

 On the other hand, as an alternative method that is simpler and applicable to the steady-state method, several authors have proposed non-invasive ^15^O-gas PET using the so-called reference region method based on the steady-state method and other acquisition methods (19-21), which calculates relative values of each hemodynamic parameter to a user-defined reference region instead of calculating absolute values. These reference region-based methods can detect elevated relative OEF values in the ipsilateral hemisphere, a specific feature of stage II hemodynamic changes in patients with occlusive cerebrovascular disease. However, these existing methods do not perform the blood volume correction usually required for quantitative ^15^O-gas PET (19) or only perform simple correction using CBV assumptions or pre-determined adjustment parameters (20, 21). Thus, with the existing methods, the reference region-based relative OEF estimates inevitably include errors due to CBV, which is a problem considering CBV is much higher in the ipsilateral hemisphere with stage II.

 This study proposes a non-invasive, clinically realistic method for measuring relative values of CBF, CBV, OEF, and CMRO_2_ by ^15^O-gas PET with the SS protocol. The proposed method extends the reference region-based method we developed for ^15^O-PET with the short inhalation protocol (20) to the steady-state method protocol. It also applies direct vascular component correction using C^15^O images to improve the accuracy of OEF estimation. To demonstrate the clinical applicability of this method, we conducted a direct comparison with the results of conventional quantitative analysis using the blood sampling data in patients with steno-occlusive lesions of major cerebral arteries (n=8) performed on an integrated PET/MRI scanner. The proposed method is a reference region-based method; it is necessary to define a reference region in a normal hemodynamic condition in advance and set values for each hemodynamic parameter, CBF, CBV, and OEF in the reference region. Therefore, the discrepancy between these assumed values and the actual values in each patient is an error source for each parameter map; the degree of error was evaluated by computer simulation assuming some hemodynamic scenarios.

## Methods

### Theory

The theory of the present method for quantification of CBF, CBV, OEF, and CMRO_2_ using PET and oxygen-15 labeled gases without an arterial blood sampling based on the steady-state method (see appendix) (17, 18, 22) is as follows. CBF, CBV, OEF, and CMRO_2_ in a brain region are defined as${\mathrm{CBF}}_{i}$, ${\mathrm{CBV}}_{i}$, ${\mathrm{OEF}}_{i}$, and ${\mathrm{CMRO}}_{2i}$, respectively. CBF, CBV, OEF, and CMRO_2_ in the reference brain region are defined as ${\mathrm{CBF}}_{\mathrm{Ref}}$, ${\mathrm{CBV}}_{\mathrm{Ref}}$, ${\mathrm{OEF}}_{\mathrm{Ref}}$, and ${\mathrm{CMRO}}_{2\mathrm{Ref}}$, respectively. The radioactivity concentrations in the brain and blood are defined as follows:

$C_{i}^{\mathrm{CO2}}$ : Radioactivity concentration of H_2_^15^O in a brain region measured by PET during inhalation of C^15^O_2_ gas after equilibrium had been reached

$C_{i}^{\mathrm{CO}}$ : Radioactivity concentration of hemoglobin (Hb)C^15^O in a brain region measured by PET after inhalation of C^15^O gas

$C_{i}^{\mathrm{O2}}$ : Radioactivity concentration in a brain region measured by PET during inhalation of ^15^O_2_ gas after equilibrium had been reached

$C_{\mathrm{Ref}}^{\mathrm{CO2}}$ : Radioactivity concentration of H_2_^15^O in the reference brain region measured by PET during inhalation of C^15^O_2_ gas after equilibrium

had been reached

$C_{\mathrm{Ref}}^{\mathrm{CO}}$ : Radioactivity concentration of HbC^15^O in the reference brain region measured by PET after inhalation of C^15^O gas

$C_{\mathrm{Ref}}^{\mathrm{O2}}$ :Radioactivity concentration in the reference brain region measured by PET during inhalation of ^15^O_2_ gas after equilibrium had been reached

$C_{a}^{\mathrm{CO2}}$ : Radioactivity concentration of H_2_^15^O in whole blood during inhalation of C^15^O_2_ gas after equilibrium had been reached

$C_{p}^{\mathrm{CO2}}$ : Radioactivity concentration of H_2_^15^O in plasma during inhalation of C^15^O_2_ gas after equilibrium had been reached

$C_{a}^{\mathrm{CO}}$ : Radioactivity concentration of HbC^15^O in whole blood after inhalation of C^15^O gas

$C_{a}^{\mathrm{O2}}$ : Radioactivity concentration in whole blood during inhalation of ^15^O_2_ gas after equilibrium had been reached

$C_{a[o]}^{\mathrm{O2}}$ : Radioactivity concentration of Hb^15^O_2_ in whole blood during inhalation of ^15^O_2_ gas after equilibrium had been reached

$C_{a[w]}^{\mathrm{O2}}$ : Radioactivity concentration of H_2_^15^O in whole blood during inhalation of ^15^O_2_ gas after equilibrium had been reached

$C_{p[w]}^{\mathrm{O2}}$ : Radioactivity concentration of H_2_^15^O in plasma during inhalation of ^15^O_2_ gas after equilibrium had been reached

 The ratios of values in a brain region to the reference region for CBF, CBV, OEF, and CMRO_2_ are defined as ${\mathrm{RCBF}}_{i}$, ${\mathrm{RCBV}}_{i}$, ${\mathrm{ROEF}}_{i}$, and ${\mathrm{RCMRO}}_{2i}$, respectively as follows:

${\mathrm{RCBF}}_{i}=\frac{{\mathrm{CBF}}_{i}}{{\mathrm{CBF}}_{\mathrm{Ref}}}$ Eq. 1

${\mathrm{RCBV}}_{i}=\frac{{\mathrm{CBV}}_{i}}{{\mathrm{CBV}}_{\mathrm{Ref}}}$ Eq. 2

${\mathrm{ROEF}}_{i}=\frac{{\mathrm{OEF}}_{i}}{{\mathrm{OEF}}_{\mathrm{Ref}}}$ Eq. 3

${\mathrm{RCMRO}}_{2i}=\frac{{\mathrm{CMRO}}_{2i}}{{\mathrm{CMRO}}_{2\mathrm{Ref}}}$ Eq. 4

**Figure 1:**
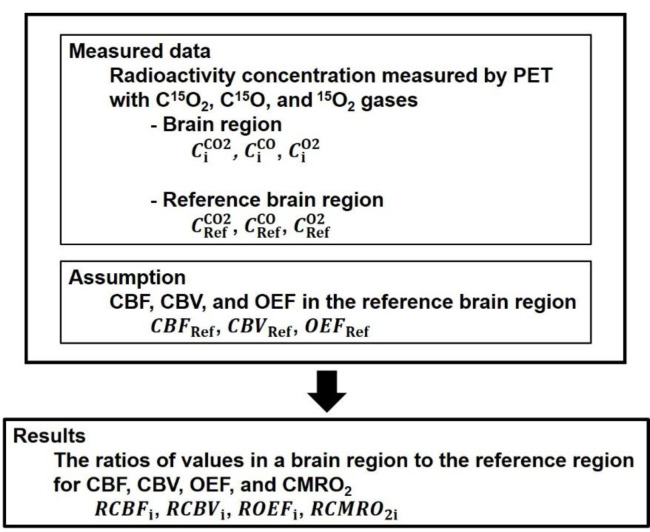
The flowchart for calculation of the ratios of values in a brain region to the reference region for CBF, CBV, OEF, and CMRO_2_ (${\mathrm{RCBF}}_{i}$, ${\mathrm{RCBV}}_{i}$, ${\mathrm{ROEF}}_{i}$, and ${\mathrm{RCMRO}}_{2i}$, respectively). From radioactivity concentrations in a brain region and the reference brain region measured by PET with C^15^O_2_, C^15^O, and ^15^O_2_ gases ($C_{i}^{\mathrm{CO2}}$, $C_{i}^{\mathrm{CO}}$, $C_{i}^{\mathrm{O2}}$, $C_{\mathrm{Ref}}^{\mathrm{CO2}}$, $C_{\mathrm{Ref}}^{\mathrm{CO}}$, $C_{\mathrm{Ref}}^{\mathrm{O2}}$, respectively), and assumed values of CBF, CBV, OEF, and CMRO_2_ in the reference brain region (${\mathrm{CBF}}_{\mathrm{Ref}}$, ${\mathrm{CBV}}_{\mathrm{Ref}}$, ${\mathrm{OEF}}_{\mathrm{Ref}}$, and ${\mathrm{CMRO}}_{2\mathrm{Ref}}$ ,respectively), ${\mathrm{RCBF}}_{i}$, ${\mathrm{RCBV}}_{i}$, ${\mathrm{ROEF}}_{i}$, and ${\mathrm{RCMRO}}_{2i}$ are calculated

 In the present method, ${\mathrm{RCBF}}_{i}$, ${\mathrm{RCBV}}_{i}$, ${\mathrm{ROEF}}_{i}$, and ${\mathrm{RCMRO}}_{2i}$ were calculated without invasive arterial blood sampling. The flowchart for calculation of ${\mathrm{RCBF}}_{i}$, ${\mathrm{RCBV}}_{i}$, ${\mathrm{ROEF}}_{i}$, and ${\mathrm{RCMRO}}_{2i}$, is shown in Figure 1. From radioactivity concentrations in a brain region and the reference brain region measured by PET with C^15^O_2_, C^15^O, and ^15^O_2_ gases ($C_{i}^{\mathrm{CO2}}$, $C_{i}^{\mathrm{CO}}$, $C_{i}^{\mathrm{O2}},$ $C_{\mathrm{Ref}}^{\mathrm{CO2}}$, $C_{\mathrm{Ref}}^{\mathrm{CO}}$, $C_{\mathrm{Ref}}^{\mathrm{O2}}$, respectively), and assumed values of CBF, CBV, OEF, and CMRO_2_ in the reference brain region (${\mathrm{CBF}}_{\mathrm{Ref}}$, ${\mathrm{CBV}}_{\mathrm{Ref}}$, ${\mathrm{OEF}}_{\mathrm{Ref}}$, and ${\mathrm{CMRO}}_{2\mathrm{Ref}}$,respectively), ${\mathrm{RCBF}}_{i}$, ${\mathrm{RCBV}}_{i}$, ${\mathrm{ROEF}}_{i}$, and ${\mathrm{RCMRO}}_{2i}$ are calculated.

***1*** ***.*** ***Calculation of*** ${\mathrm{RCBF}}_{i}$

 The following equations are derived from Eq. A2:

$C_{\mathrm{Ref}}^{\mathrm{CO2}}=C_{a}^{\mathrm{CO2}}∙\frac{{\mathrm{CBF}}_{\mathrm{Ref}}}{\frac{{\mathrm{CBF}}_{\mathrm{Ref}}}{p}+\lambda }$ Eq. 5

 Where *p* is the brain-blood partition coefficient, which can be assumed to be unity.   is the decay constant of ^15^O. When ${\mathrm{CBF}}_{\mathrm{Ref}}$is assumed, $C_{a}^{\mathrm{CO2}}$ can be calculated. The following equations are derived from Eq. A3:

${\mathrm{CBF}}_{i}=\frac{\lambda }{\frac{C_{a}^{\mathrm{CO2}}}{C_{i}^{\mathrm{CO2}}}-\frac{1}{p}}$ Eq. 6

 Substituting $C_{a}^{\mathrm{CO2}}$into Eq. 6 yields ${\mathrm{CBF}}_{i}$ for$C_{i}^{\mathrm{CO2}}$, and Eq. 1 yields ${\mathrm{RCBF}}_{i}$.

***2*** ***.*** ***Calculation of*** ${\mathrm{RCBV}}_{i}$

 The following equations are derived from Eq. A4:

$C_{\mathrm{Ref}}^{\mathrm{CO}}=C_{a}^{\mathrm{CO}}∙{\mathrm{CBV}}_{\mathrm{Ref}}∙\frac{h}{H}$ Eq. 7

 Where h and H are hematocrits in the cerebral and large vessels, respectively. The hematocrit ratio of cerebral to large vessels*, h/(H* ), can be assumed to be 0.85 (18, 23, 24). When ${\mathrm{CBV}}_{\mathrm{Ref}}$is assumed, $C_{a}^{\mathrm{CO}}$can be calculated. The following equations are derived from Eq. A5:

${\mathrm{CBV}}_{i}=\frac{C_{i}^{\mathrm{CO}}}{C_{a}^{\mathrm{CO}}}∙\frac{H}{h}$ Eq. 8

 Substituting $C_{a}^{\mathrm{CO}}$ into Eq. 8 yields ${\mathrm{CBV}}_{i}$for $C_{i}^{\mathrm{CO}}$, and Eq. 2 yields ${\mathrm{RCBV}}_{i}$.

***3*** ***.*** ***Calculation of*** ${\mathrm{ROEF}}_{i}$

 The following equations are derived from Eq. A9:

${\mathrm{OEF'}}_{i}=\frac{C_{a}^{\mathrm{CO2}}}{C_{a[o]}^{\mathrm{O2}}}∙\left(\frac{C_{i}^{\mathrm{O2}}}{C_{i}^{\mathrm{CO2}}}-\frac{C_{a\left[w\right]}^{\mathrm{O2}}}{C_{a}^{\mathrm{CO2}}}\right)=\frac{\frac{C_{i}^{\mathrm{O2}}}{C_{a[o]}^{\mathrm{O2}}}}{\frac{C_{i}^{\mathrm{CO2}}}{C_{a}^{\mathrm{CO2}}}}-R_{m}$ Eq. 9

Or

$C_{i}^{\mathrm{O2}}=C_{a[o]}^{\mathrm{O2}}∙\frac{C_{i}^{\mathrm{CO2}}}{C_{a}^{\mathrm{CO2}}}∙\left({\mathrm{OEF'}}_{i}+R_{m}\right)$ Eq. 9’

Where

$R_{m}=\frac{C_{a[w]}^{\mathrm{O2}}}{C_{a[o]}^{\mathrm{O2}}}$ Eq. 10

 R_m_ can be assumed to be 0.214 (25). ${\mathrm{OEF'}}_{i}$is cerebral oxygen extraction fraction without correction of remaining radioactivity concentration of ^15^O_2_ in the cerebral vessels (26, 27). The relation between ${\mathrm{OEF'}}_{i}$and cerebral oxygen extraction fraction with correction of remaining radioactivity concentration of ^15^O_2_ in the cerebral vessels, ${\mathrm{OEF'}}_{i}$, can be expressed as follows (Eq. A13):

${\mathrm{OE}F^{'}}_{i}={\mathrm{OEF}}_{i}∙\left(1-X_{i}\right)+X_{i}$ Eq. 11

Or

${\mathrm{OEF}}_{i}=\frac{{\mathrm{OEF'}}_{i}-X_{i}}{1-X_{i}}$ Eq. 11’

Where (Eq. A14)

$X_{i}=\frac{\frac{{\mathrm{CBF}}_{i}}{p}+}{\frac{{\mathrm{CBF}}_{i}}{\frac{h}{H}{\mathrm{CBV}}_{i}}+}$ Eq. 12

 The following equations are derived from Eq. 9’, 11, and 12:

$C_{\mathrm{Ref}}^{\mathrm{O2}}=C_{a[o]}^{\mathrm{O2}}∙\frac{C_{\mathrm{Ref}}^{\mathrm{CO2}}}{C_{a}^{\mathrm{CO2}}}∙\left({\mathrm{OEF'}}_{\mathrm{Ref}}+R_{m}\right)$ Eq. 13

${\mathrm{OE}F^{'}}_{\mathrm{Ref}}={\mathrm{OEF}}_{\mathrm{Ref}}∙\left(1-X_{\mathrm{Ref}}\right)+X_{\mathrm{Ref}}$ Eq. 14

$X_{\mathrm{Ref}}=\frac{\frac{{\mathrm{CBF}}_{\mathrm{Ref}}}{p}+}{\frac{{\mathrm{CBF}}_{\mathrm{Ref}}}{\frac{h}{H}{\mathrm{CBV}}_{\mathrm{Ref}}}+}$ Eq. 15

 When ${\mathrm{OEF}}_{\mathrm{Ref}}$is assumed, $C_{a[o]}^{\mathrm{O2}}$can be calculated using ${\mathrm{CBF}}_{\mathrm{Ref}}$, ${\mathrm{CBV}}_{\mathrm{Ref}}$, $C_{\mathrm{Ref}}^{\mathrm{CO2}}$, $C_{\mathrm{Ref}}^{\mathrm{O2}}$, and $C_{a}^{\mathrm{CO2}}$. ${\mathrm{OEF}}_{i}$can be calculated from Eq, 9, 11’, and 12, and Eq. 3 yields ${\mathrm{ROEF}}_{i}$.

***4*** ***.*** ***Calculation of*** ${\mathrm{RCMRO}}_{2i}$

 The following equations are derived from Eq. A15:

${\mathrm{CMRO}}_{2i}={\mathrm{OEF}}_{i}∙{\mathrm{CBF}}_{i}∙[O_{2}]$ Eq. 16

${\mathrm{CMRO}}_{2\mathrm{Ref}}={\mathrm{OEF}}_{\mathrm{Ref}}∙{\mathrm{CBF}}_{\mathrm{Ref}}∙[O_{2}]$ Eq. 17

 Where [O_2_] is the total oxygen content in arterial blood. The following equation is derived from Eq. 4, 16, and 17.

${\mathrm{RCMRO}}_{2i}=\frac{{\mathrm{CMRO}}_{2i}}{{\mathrm{CMRO}}_{2\mathrm{Ref}}}=\frac{{\mathrm{OEF}}_{i}∙{\mathrm{CBF}}_{i}}{{\mathrm{OEF}}_{\mathrm{Ref}}∙{\mathrm{CBF}}_{\mathrm{Ref}}}$ Eq. 18

${\mathrm{RCMRO}}_{2i}$ can be calculated using ${\mathrm{CBF}}_{i}$, ${\mathrm{OEF}}_{i}$, ${\mathrm{CBF}}_{\mathrm{Ref}}$, and ${\mathrm{OEF}}_{\mathrm{Ref}}$.

 In the present method, ${\mathrm{CBF}}_{\mathrm{Ref}}$, ${\mathrm{CBV}}_{\mathrm{Ref}}$, and ${\mathrm{OEF}}_{\mathrm{Ref}}$ were assumed to be 0.3 mL/mL/min, 0.03 mL/mL, and 0.4, respectively (22).

### Subjects

 This study is a retrospective analysis of consecutive PET examinations using oxygen-15 labeled gases performed on eight patients with steno-occlusive lesions of major cerebral arteries (49-67 years of age; 5 males and 3 females; 1 internal carotid artery stenosis, 3 middle cerebral artery stenosis, 4 middle cerebral artery occlusion) between April 2018 and December 2022. The study was approved by the Institutional Review Board of Fukushima Medical University, Fukushima, Japan.

### PET Experimental Procedure

 All PET studies were performed with a Siemens mMR PET/MRI scanner, which provides 127 sections with an axial field of view of 25.8 cm (28). The intrinsic spatial resolution was 4.3 mm full-width at half maximum (FWHM) in-plane and 4.3 mm FWHM axially. 

 Data were acquired in three-dimensional mode. Scatter was corrected (29). PET measurements with the steady-state method of ^15^O-labeled gases, C^15^O, ^15^O_2_, and C^15^O_2_ were performed on all subjects (17, 18, 22). Static PET scanning was started 3 min after 1 min of continuous inhalation of C^15^O gas (a total of approximately 3 GBq supplied by mouth). The scanning time was 4 min. Then, static PET scanning was performed during inhalation of ^15^O_2_ gas after equilibrium had been reached and confirmed by the head radioactivity curve (a total of approximately 8.4 GBq supplied by mouth). The scanning time was 10 min, and the time from the beginning of inhalation to the beginning of scanning was 10 min. Static PET scanning was performed during inhalation of C^15^O_2_ gas using the same protocol as that used with ^15^O_2_ gas (a total of approximately 2.8 GBq supplied by mouth). During each PET scanning, arterial blood sampling was performed to measure the radioactivity concentration in the blood and plasma. Arterial blood gases were also measured. Total oxygen content in arterial blood was estimated from P_a_O_2_, pH, and hemoglobin (Hb) concentration (18, 30). PET image reconstruction was carried out by the ordered-subset expectation maximization (OSEM) algorithm (iterations: 3, subsets: 21) with a post-reconstruction Gaussian filter of 5 mm FWHM. Using reconstructed PET images, the radioactivity concentration in arterial blood and plasma, and arterial blood gases data, the parametric images of CBF, CBV, OEF, and CMRO_2_ were calculated (See appendix) (17).

 All MRI studies were performed with a Siemens mMR PET/MRI scanner, equipped with a 3.0-T MR scanner, during and between PET scanning. Dixon sequence (DIXON) (3D-VIBE (volumetric interpolated breath-hold examination), TR: 3.56 ms, TE: 1.23 ms and 2.46 ms; field of view: 500 mm, slice thickness: 3.12 mm, resolution: 2.6×2.6×3.1 mm, 1 slab: 128 slices) was performed for attenuation correction of PET (31). Three-dimensional volumetric T1-weighted images (T1WI) and T2-weighted images (T2WI), diffusion-tensor images, arterial spin labeling images, and MR angiography were also acquired.

### Data analysis

 Regions of interest (ROIs) were drawn on all PET images, referring to T1WI and T2WI. Circular ROIs (10 mm in diameter) were defined for the area of lesion in the cerebrum, excluding cerebral infarction. ROIs were also defined for the contralateral side of the cerebral lesion and the ipsilateral cerebellar cortex of the cerebral lesion. The ipsilateral cerebellar cortex of the cerebral lesion was used as the reference brain region. The ratio of CBF, CBV, OEF, and CMRO_2_ with arterial blood sampling based on the steady-state method in the cerebral lesion and its contralateral side to those in the reference brain region were calculated. These values were compared with${\mathrm{RCBF}}_{i}$ ${\mathrm{RCBV}}_{i}$ ${\mathrm{ROEF}}_{i}$ and ${\mathrm{RCMRO}}_{2i},$ calculated by the present method.

### Simulation studies

 Simulation studies were performed to estimate systematic errors of ${\mathrm{RCBF}}_{i}$, ${\mathrm{ROEF}}_{i}$, and ${\mathrm{RCMRO}}_{2i}$ calculated by the present method when CBF in the reference brain region, ${\mathrm{CBF}}_{\mathrm{Ref}}$, deviates from the assumed value. The assumed values of ${\mathrm{CBF}}_{\mathrm{Ref}}$, ${\mathrm{CBV}}_{\mathrm{Ref}}$, and ${\mathrm{OEF}}_{\mathrm{Ref}}$ were 0.3 mL/mL/min, 0.03 mL/mL, and 0.4, respectively. ${\mathrm{CBF}}_{\mathrm{Ref}}$ was varied in five steps from -30% to +30% from the assumed value. ${\mathrm{CBV}}_{\mathrm{Ref}}$ and${\mathrm{OEF}}_{\mathrm{Ref}}$ were also varied so that ${\mathrm{CBV}}_{\mathrm{Ref}}$ divided by${\mathrm{CBF}}_{\mathrm{Ref}}$, corresponding to vascular mean transit time, and ${\mathrm{CBF}}_{\mathrm{Ref}}$ multiplied by ${\mathrm{OEF}}_{\mathrm{Ref}}$, corresponding to ${\mathrm{CMRO}}_{2\mathrm{Ref}}$, were constant.

### Errors of RCBF_i

 CBF often decreases in occlusive cerebrovascular diseases. Thus, the systematic error of ${\mathrm{RCBF}}_{i}$ was estimated when ${\mathrm{RCBF}}_{i}$ varied from 0.2 to 1.0. ${\mathrm{RCBF}}_{i}$ values were estimated using radioactivity concentrations in a brain region and the reference brain region, which were calculated according to the assumptions. The estimated ${\mathrm{RCBF}}_{i}$ values were compared to the assumed ${\mathrm{RCBF}}_{i}$ values.

### Errors of ROEF_i

 The stage II hemodynamic change in occlusive cerebrovascular diseases due to decreased cerebral perfusion pressure below the lower limit of cerebral autoregulation shows a decrease in CBF with an increase in OEF for maintenance of CMRO_2_, so-called “misery perfusion” (2). Thus, the systematic error of ${\mathrm{ROEF}}_{i}$ was estimated when ${\mathrm{ROEF}}_{i}$ varied from 1.0 to 2.0. In this simulation, ${\mathrm{CBF}}_{i}$ and ${\mathrm{OEF}}_{i}$ were varied so that ${\mathrm{CBF}}_{i}$ multiplied by${\mathrm{OEF}}_{i}$, corresponding to${\mathrm{CMRO}}_{2i}$, were constant. Since the stage II hemodynamic change also shows an increase in CBV (2), ${\mathrm{RCBV}}_{i}$ was varied to 1.0, 1.5, and 2.0. ${\mathrm{ROEF}}_{i}$ values were estimated using radioactivity concentrations in a brain region and the reference brain region, which were calculated according to the assumptions. The estimated ${\mathrm{ROEF}}_{i}$ values were compared to the assumed ${\mathrm{ROEF}}_{i}$ values.

### Errors of RCMRO_2i

Oxygen hypometabolism with hypoperfusion in occlusive cerebrovascular diseases can be observed in lesions with neural damage, so called “matched hypoperfusion” (32). Thus, the systematic error of ${\mathrm{RCMRO}}_{2i}$ was estimated when ${\mathrm{RCMRO}}_{2i}$ varied from 0.2 to 1.0. ${\mathrm{CBF}}_{i}$ and ${\mathrm{CMRO}}_{2i}$ were varied so that ${\mathrm{ROEF}}_{i}$ and ${\mathrm{RCBV}}_{i}$ divided by ${\mathrm{RCBF}}_{i}$, corresponding to vascular mean transit time, were unity (33). ${\mathrm{RCMRO}}_{2i}$values were estimated using radioactivity concentrations in a brain region and the reference brain region, which were calculated according to the assumptions. The estimated ${\mathrm{RCMRO}}_{2i}$ values were compared to the assumed ${\mathrm{RCMRO}}_{2i}$ values.

## Results

**Figure 2:**
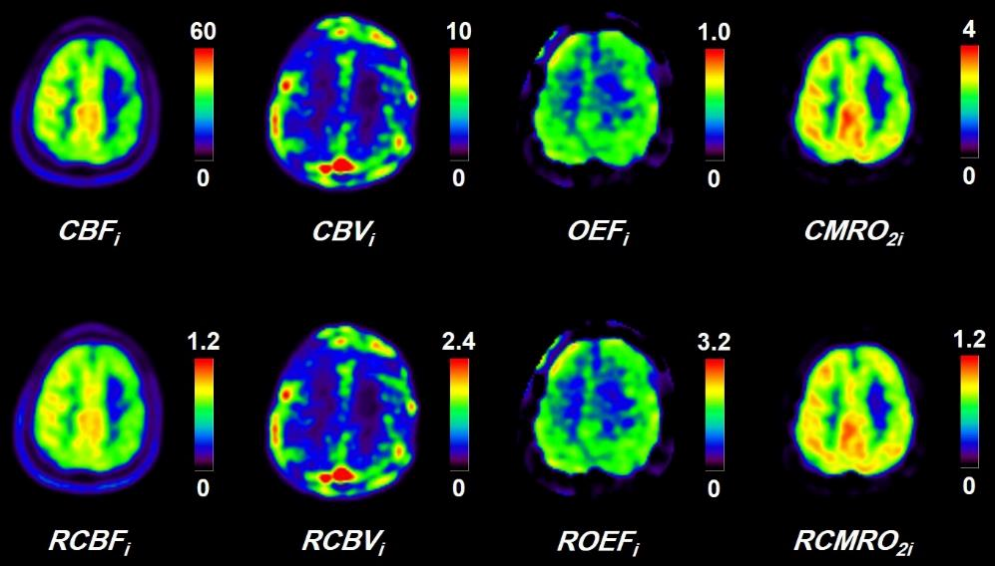
Typical PET images of ${\mathrm{CBF}}_{i}$, ${\mathrm{CBV}}_{i}$, ${\mathrm{OEF}}_{i}$, and ${\mathrm{CMRO}}_{2i}$ obtained by the steady-state method with arterial blood sampling and corresponding PET images of ${\mathrm{RCBF}}_{i}$, ${\mathrm{RCBV}}_{i}$, ${\mathrm{ROEF}}_{i}$, and ${\mathrm{RCMRO}}_{2i}$ calculated by the present method for a patient with left middle cerebral artery occlusion. Scale maximum values are 60 mL/100 mL/min, 10 mL/100 mL, 1.0, and 4 mL/100 mL/min for ${\mathrm{CBF}}_{i}$, ${\mathrm{CBV}}_{i}$, ${\mathrm{OEF}}_{i}$, and ${\mathrm{CMRO}}_{2i}$ images, respectively

Figure 2 shows typical PET images of ${\mathrm{CBF}}_{i}$, ${\mathrm{CBV}}_{i}$, ${\mathrm{OEF}}_{i}$, and ${\mathrm{CMRO}}_{2i}$ obtained by the steady-state method with arterial blood sampling and corresponding PET images of ${\mathrm{RCBF}}_{i}$, ${\mathrm{RCBV}}_{i}$, ${\mathrm{ROEF}}_{i}$, and ${\mathrm{RCMRO}}_{2i}$ calculated by the present method for a patient with left middle cerebral artery occlusion. Similar regional distributions were observed between them.

**Figure 3:**
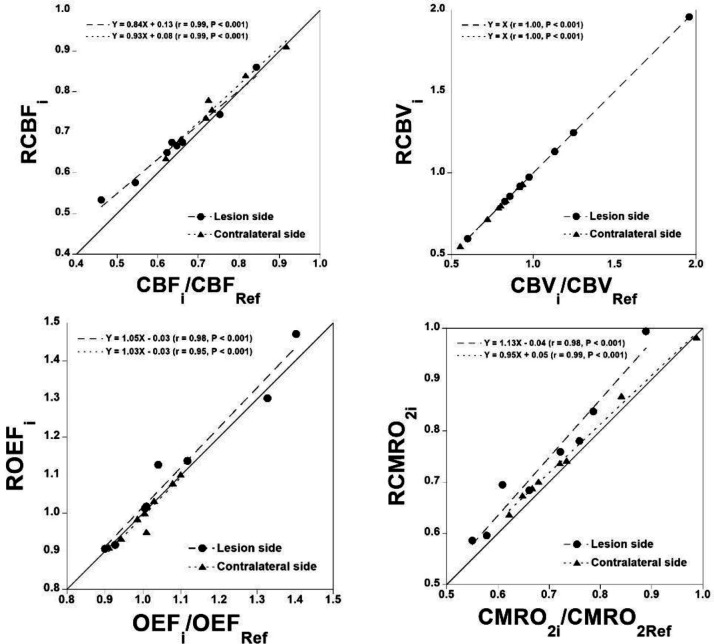
The relation between the ratio of ${\mathrm{CBF}}_{i}$ to ${\mathrm{CBF}}_{\mathrm{Ref}}$ obtained by the steady-state method with arterial blood sampling and ${\mathrm{RCBF}}_{i}$ calculated by the present method for both the cerebral lesion and the contralateral side of the cerebral lesion (**A**). Such relations for ${\mathrm{RCBV}}_{i}$, ${\mathrm{ROEF}}_{i}$, and ${\mathrm{RCMRO}}_{2i}$ are also shown (**B**, **C**, and **D**, respectively)

 The relation between the ratio of ${\mathrm{CBF}}_{i}$ to ${\mathrm{CBF}}_{\mathrm{Ref}}$ obtained by the steady-state method with arterial blood sampling and ${\mathrm{RCBF}}_{i}$ calculated by the present method for both the cerebral lesion and the contralateral side of the cerebral lesion is shown in Figure 3A. Such relations for${\mathrm{RCBV}}_{i}$,${\mathrm{ROEF}}_{i}$ and ${\mathrm{RCMRO}}_{2i}$ are also demonstrated in Figure 3B, 3C, and 3D, respectively. Good correlations were observed for all relations.

**Figure 4:**
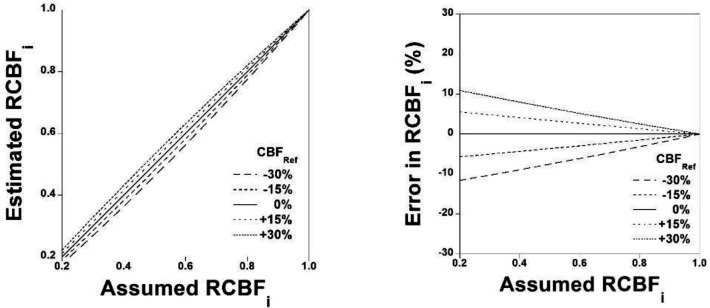
Simulation of relation between the assumed ${\mathrm{RCBF}}_{i}$ and estimated ${\mathrm{RCBF}}_{i}$ when ${\mathrm{CBF}}_{\mathrm{Ref}}$ deviates from the assumed value (**A**). Simulation of errors in the estimated ${\mathrm{RCBF}}_{i}$ for assumed ${\mathrm{RCBF}}_{i}$ (**B**)

 The simulation of relations between assumed ${\mathrm{RCBF}}_{i}$ and estimated ${\mathrm{RCBF}}_{i}$ when ${\mathrm{CBF}}_{\mathrm{Ref}}$ deviates from the assumed value is shown in Figure 4A. The simulation of errors in estimated ${\mathrm{RCBF}}_{i}$ for assumed ${\mathrm{RCBF}}_{i}$ is also shown in Figure 4B. Errors in estimated ${\mathrm{RCBF}}_{i}$ were within ±10% when assumed ${\mathrm{RCBF}}_{i}$ varied from 0.4 to 1.0.

**Figure 5:**
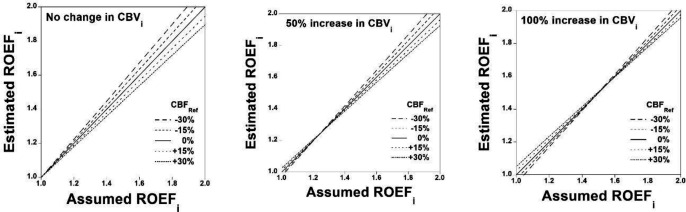
Simulation of relation between the assumed ${\mathrm{ROEF}}_{i}$ and estimated ${\mathrm{ROEF}}_{i}$ when ${\mathrm{CBF}}_{\mathrm{Ref}}$ deviates from the assumed value for ${\mathrm{RCBV}}_{i}$ of 1.0, 1.5, and 2.0 (**A**, **B**, and **C**, respectively)

**Figure 6:**
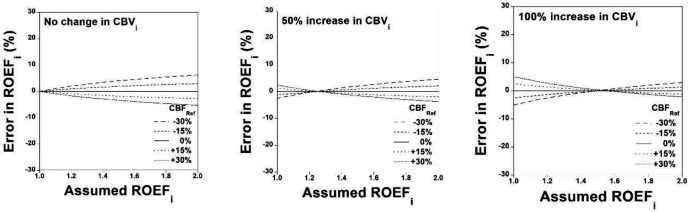
Simulation of errors in the estimated ${\mathrm{ROEF}}_{i}$ for assumed ${\mathrm{ROEF}}_{i}$ for ${\mathrm{RCBV}}_{i}$ of 1.0, 1.5, and 2.0 (**A**, **B**, and **C**, respectively)

 The simulation of relations between assumed ${\mathrm{ROEF}}_{i}$ and estimated ${\mathrm{ROEF}}_{i}$ when ${\mathrm{CBF}}_{\mathrm{Ref}}$ deviates from the assumed value is shown in Figure 5A, 5B, and 5C for ${\mathrm{RCBV}}_{i}$ of 1.0, 1.5, and 2.0, respectively. The simulation of errors in estimated ${\mathrm{ROEF}}_{i}$ for assumed ${\mathrm{ROEF}}_{i}$ is also shown in Figure 6A, 6B, and 6C for ${\mathrm{RCBV}}_{i}$ of 1.0, 1.5, and 2.0, respectively. Errors in estimated ${\mathrm{ROEF}}_{i}$ were within ±5% when assumed ${\mathrm{ROEF}}_{i}$ varied from 1.0 to 1.5.

**Figure 7:**
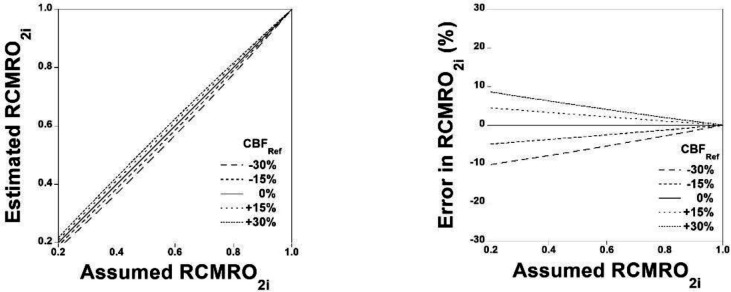
Simulation of relation between the assumed ${\mathrm{RCMRO}}_{2i}$ and estimated ${\mathrm{RCMRO}}_{2i}$ when ${\mathrm{CBF}}_{\mathrm{Ref}}$ deviates from the assumed value (**A**). Simulation of errors in the estimated ${\mathrm{RCMRO}}_{2i}$ for assumed ${\mathrm{RCMRO}}_{2i}$ (**B**)

 The simulation of relations between assumed ${\mathrm{RCMRO}}_{2i}$and estimated ${\mathrm{RCMRO}}_{2i}$ when ${\mathrm{CBF}}_{\mathrm{Ref}}$ deviates from the assumed value is shown in Figure 7A. The simulation of errors in estimated ${\mathrm{RCMRO}}_{2i}$ for assumed ${\mathrm{RCMRO}}_{2i}$ is also shown in Figure 7B. Errors in estimated ${\mathrm{RCMRO}}_{2i}$ were within ±10% when assumed ${\mathrm{RCMRO}}_{2i}$ varied from 0.4 to 1.0.

## Discussion

The measurement of CBF, CBV, OEF, and CMRO_2_ using PET and oxygen-15 labeled gases is widely used to investigate the pathophysiology of occlusive cerebrovascular disease (1-8). The steady-state method for quantification of CBF, CBV, OEF, and CMRO_2_ is a method that has been commonly used for a long time (17, 18, 22). However, this method requires invasive arterial blood sampling. Therefore, we have developed a method for quantification of CBF, CBV, OEF, and CMRO_2_ without invasive arterial blood sampling based on the steady-state method which is simple in PET examination procedure and needs the long PET examination time as compared with other various acquisition protocols.

 In the present study, good correlations were observed for ${\mathrm{RCBF}}_{i}$, ${\mathrm{RCBV}}_{i}$, ${\mathrm{ROEF}}_{i}$, and ${\mathrm{RCMRO}}_{2i}$ calculated by the present method compared with those obtained by the steady-state method with arterial blood sampling for both the cerebral lesions of occlusive cerebrovascular diseases and the contralateral side of cerebral lesions. The simulation showed that errors in estimated ${\mathrm{RCBF}}_{i}$ were within ±10% when assumed ${\mathrm{RCBF}}_{i}$ varied from 0.4 to 1.0. These results indicate the validity of the present method.

 The stage II hemodynamic change in occlusive cerebrovascular diseases, so-called “misery perfusion”, due to decreased cerebral perfusion pressure below the lower limit of cerebral autoregulation shows a decrease in CBF with an increase in OEF for maintenance of CMRO_2_ and with an increase in CBV (2). In the simulation studies, errors in estimated ${\mathrm{ROEF}}_{i}$ were within ±5% when assumed ${\mathrm{ROEF}}_{i}$ varied from 1.0 to 1.5 for ${\mathrm{RCBV}}_{i}$ of 1.0 to 2.0, indicating the validity of the present method in the condition of misery perfusion. To estimate OEF, an indicator of stage II hemodynamic change, some non-invasive methods have been developed that used PET scanning data with ^15^O-labeled water and ^15^O_2_ gas but without C^15^O gas (19, 20). However, increased CBV in stage II hemodynamic change might cause non-negligible OEF estimation errors (20). In the present method, ${\mathrm{RCBV}}_{i}$ was also estimated using PET scanning data with C^15^O gas; therefore, errors in estimated ${\mathrm{ROEF}}_{i}$ were negligibly small.

 Oxygen hypometabolism with hypoperfusion in occlusive cerebrovascular diseases, so-called “matched hypoperfusion”, showing decreases in CBF and CMRO_2_ can be observed in lesions with neural damage (32). In the simulation studies, errors in estimated ${\mathrm{RCMRO}}_{2i}$ were within ±10% when assumed ${\mathrm{RCMRO}}_{2i}$ varied from 0.4 to 1.0, indicating the validity of the present method in the condition of matched hypoperfusion.

 Good correlations were observed for ${\mathrm{RCBF}}_{i}$, ${\mathrm{RCBV}}_{i}$, ${\mathrm{ROEF}}_{i}$, and ${\mathrm{RCMRO}}_{2i}$ calculated by the present method compared to those obtained by the steady-state method with arterial blood sampling. Simulation studies showed that errors in estimated ${\mathrm{RCBF}}_{i}$, ${\mathrm{ROEF}}_{i}$, and ${\mathrm{RCMRO}}_{2i}$ were negligibly small. However, a limitation of this study is the small number of patients (n=8). The present method should be validated with large series of patients in a multicenter study with various PET scanners and reconstruction methods.

## Conclusion

 A simple method for quantification of CBF, CBV, OEF, and CMRO_2_ using PET and three oxygen-15 labeled gases, C^15^O_2_, C^15^O, and ^15^O_2_, without invasive arterial blood sampling has been developed based on the steady-state method. Good correlations were observed for ${\mathrm{RCBF}}_{i}$, ${\mathrm{RCBV}}_{i}$, ${\mathrm{ROEF}}_{i}$, and ${\mathrm{RCMRO}}_{2i}$ calculated by the present method compared with those obtained by the steady-state method with arterial blood sampling, indicating its validity. Simulation studies showed that errors in estimated ${\mathrm{RCBF}}_{i}$, ${\mathrm{ROEF}}_{i}$, and ${\mathrm{RCMRO}}_{2i}$ were negligibly small for both conditions of misery perfusion and matched hypoperfusion. The present method can be used to investigate the pathophysiology of occlusive cerebrovascular disease.
